# Synergistic Effect of Pleuromutilins with Other Antimicrobial Agents against *Staphylococcus aureus In Vitro* and in an Experimental *Galleria mellonella* Model

**DOI:** 10.3389/fphar.2017.00553

**Published:** 2017-08-22

**Authors:** Chun-Liu Dong, Lin-Xiong Li, Ze-Hua Cui, Shu-Wen Chen, Yan Q. Xiong, Jia-Qi Lu, Xiao-Ping Liao, Yuan Gao, Jian Sun, Ya-Hong Liu

**Affiliations:** ^1^National Risk Assessment Laboratory for Antimicrobial Resistance of Animal Original Bacteria, South China Agricultural University Guangzhou, China; ^2^Guangdong Provincial Key Laboratory of Veterinary Pharmaceutics Development and Safety Evaluation, South China Agricultural University Guangzhou, China; ^3^David Geffen School of Medicine, University of California, Los Angeles, Los Angeles CA, United States; ^4^Laboratory of Veterinary Pharmacology, College of Veterinary Medicine, South China Agricultural University Guangzhou, China; ^5^Jiangsu Co-Innovation Center for Prevention and Control of Important Animal Infectious Diseases and Zoonoses Yangzhou, China

**Keywords:** pleuromutilins, other antibiotics, antibiotic combination, *Staphylococcus aureus*, *Galleria mellonella* model

## Abstract

Invasive infections due to *Staphylococcus aureus*, including methicillin-resistant *S. aureus* are prevalent and life-threatening. Combinations of antibiotic therapy have been employed in many clinical settings for improving therapeutic efficacy, reducing side effects of drugs, and development of antibiotic resistance. Pleuromutilins have a potential to be developed as a new class of antibiotics for systemic use in humans. In the current study, we investigated the relationship between pleuromutilins, including valnemulin, tiamulin, and retapamulin, and 13 other antibiotics representing different mechanisms of action, against methicillin-susceptible and -resistant *S. aureus* both *in vitro* and in an experimental *Galleria mellonella* model. *In vitro* synergistic effects were observed in combination of all three study pleuromutilins with tetracycline (TET) by standard checkerboard and/or time-kill assays. In addition, the combination of pleuromutilins with ciprofloxacin or enrofloxacin showed antagonistic effects, while the rest combinations presented indifferent effects. Importantly, all study pleuromutilins in combination with TET significantly enhanced survival rates as compared to the single drug treatment in the *G. mellonella* model caused by *S. aureus* strains. Taken together, these results demonstrated synergy effects between pleuromutilins and TET against *S. aureus* both *in vitro* and *in vivo*.

## Introduction

*Staphylococcus aureus* is a predominant cause of community-acquired and healthcare-associated infection in human (VanEperen and Segreti, 2016). In particular, it is the most common cause of life-threatening endovascular infections. Despite modern antibiotic treatment, morbidity and mortality with such syndromes remain unacceptably high (Boucher and Sakoulas, 2007; Boucher et al., 2010). In addition, the growing population in methicillin-resistant *S. aureus* (MRSA) infections and the dwindling industry investment in anti-infective development further portend a looming threat of untreatable infections (Deng et al., 2017). Therefore, there is a great need to find novel strategies for the treatment of these invasive infections. One of important approaches is the combinations of preexisting antibiotics which has been addressed by our current studies.

Pleuromutilins were discovered as natural-product antibiotics in 1950s (Kavanagh et al., 1951). Tiamulin (TIA) was the first pleuromutilin compound to be approved for veterinary use in 1979, followed by valnemulin (VAL) in 1999 (Sader et al., 2012). Retapamulin (RET) became the first pleuromutilin approved for use in humans, only topical application in 2007. Recently, synthesized pleuromutilins, which combine potent antibacterial activity with favorable pharmaceutical properties, make these compounds suitable for systemic administration in humans (Zeitlinger et al., 2016). Pleuromutilins inhibit bacterial growth via a specific interaction with the 23S rRNA of the 50S bacterial ribosome subunit that is responsible for bacterial protein synthesis (Davidovich et al., 2007). Their unique mechanism of action implies a broad antibacterial spectrum against a wide range of both Gram-positive and Gram-negative bacteria (Paukner et al., 2013), including MRSA, as well a low probability of cross resistance with other antibiotics and development of resistance.

Although pleuromutilins have been used in clinical settings for almost 40 years, very little is known about their interaction with other antibiotics. Therefore, in the current study, we tested the combination of pleuromutilins with other antibiotics representing diverse mechanisms of action, including targeting protein, cell wall, DNA gyrase and folic acid syntheses against methicillin-susceptible *S. aureus* (MSSA) and MRSA *in vitro* and in an experimental *Galleria mellonella* model.

## Materials and Methods

### Antimicrobial Agents, Bacterial Strains, and Growth Conditions

Three pleuromutilins, including TIA, VAL, and RET, and 13 other antimicrobial agents were selected for our studies based on their mechanism of action (**Table 1**). All study antibiotics were purchased from Guangzhou Xiang Bo Biological Technology Co., Ltd. (Guangzhou, China). Antibiotic stocks solutions were prepared according to the manufacturer’s recommendations.

**Table 1:** Antibiotics used in this study.

Antibiotics	Abbreviation	Classification	Primary target
Cefotaxime	CTX	Cephalosporins	Cell wall
Erythromycin	ERY	Macrolides	Protein synthesis 50S
Florfenicol	FFC	Phenicols	Protein synthesis 50S
Clindamycin	CLI	Lincosamides	Protein synthesis 50S
Ciprofloxacin	CIP	Fluoroquinolones	DNA gyrase
Enrofloxacin	ENR	Fluoroquinolones	DNA gyrase
Gentamicin	GEN	Aminoglycosides	Protein synthesis 30S
Amikacin	AMK	Aminoglycosides	Protein synthesis 30S
Tetracycline	TET	Tetracyclines	Protein synthesis 30S
Valnemulin	VAL	Pleuromutilins	Protein synthesis 50S
Tiamulin	TIA	Pleuromutilins	Protein synthesis 50S
Retapamulin	RET	Pleuromutilins	Protein synthesis 50S
Vancomycin	VAN	Glycopeptides	Cell wall
Bacitracin	BCR	Polypeptides	Cell wall
Sulfamethoxazole	SMZ	Sulfonamides	Folic acid
Trimethoprim	TMP	Diaminopyrimidines	Folic acid

Two standard *S. aureus* strains (MSSA ATCC 29213 and MRSA ATCC 43300) and two *S. aureus* clinical strains (MSSA N54 and MRSA N9) were used in this study. All strains were incubated overnight at 37°C in brain heart infusion (BHI). Mueller-Hinton broth (MHB) was used for all *in vitro* susceptibility assays.

### Determination of MICs

Determination of the study antibiotic MICs was conducted by broth microdilution as recommended by the CLSI guidelines (CLSI, 2013).

### Determination of Fractional Inhibitory Concentration Index (FICI) by a Checkerboard Method

A checkerboard technique was employed to delineate the Fractional Inhibitory Concentration Index (FICI) of pleuromutilins plus antibiotic combinations (White et al., 1996). Briefly, 96 well plates containing serial dilutions of pleuromutilins + antibiotic (range, 0.125 × MIC to 4 × MIC) were inoculated with 5 × 10^5^ of *S. aureus* and incubated for 18 h. Control wells were free of pleuromutilins or antibiotic. After incubation, plates were screened for visual growth. The FICI were then calculated as previously described (≤0.5, synergy; 0.6–1.0, additivity; 1.1–4.0, indifference; >4.0, antagonism) (White et al., 1996; Odds, 2003).

### *In Vitro* Time-Kill Curves

Time-kill curves of pleuromutilins (VAL, TIA, and RET at 0.5 × MIC) and tetracycline (TET) (0.5 × MIC) alone, and in combination were carried in glass flasks containing a final inoculum of 5 × 10^5^ CFU/mL of the study *S. aureus* strain at 37°C with shaking for 24 h. At 0, 3, 6, 9, and 24 h of incubation, 0.1 mL aliquots were taken from each group, serially diluted in sterile saline, plated onto MH agar plates, and incubated at 37°C for 24 h for a viable count enumeration. All experiments were performed at least three times on different days.

### *Galleria mellonella* Model

A well-characterized *G. mellonella* model was used in this study based on previous publication (Desbois and Coote, 2011). Larvae of *G. mellonella* were obtained from Kaide Ruixin Co., Ltd. (Tianjin, China). In order to determine the optimal infection doses of the study *S. aureus* strains, *G. mellonella* larvae (∼250 mg with a creamy color) were randomly distributed in six experimental groups (*n* = 10/group), and were then infected by injection of 10 μL of logarithmic phase *S. aureus* cells (10^3^–10^9^ CFU/larval) into the last left proleg. After injection, the larvae were incubated in plastic Petri dishes at 37°C for 5 days and scored for survival daily. In all experiments, two controls were included: (1) PBS injections and (2) without any injection.

The *in vivo* efficacy of VAL, TIA, and RET alone, and in combination with TET or CIP was assessed in the same *G. mellonella* model caused by study *S. aurues* strains using the optimal infection doses as determined above (∼10^6^ CFU/larva). At 2 h post-infection, animals were randomized to receive no therapy or VAL, TIA, RET, TET, CIP alone, or VAL+TET, TIA+TET, RET+TET, VAL+CIP, TIA+CIP, or RET+CIP (*n* = 16/group). The antibiotics were administered only once (10 μL) into the last right proleg at doses of VAL, 10 mg/kg; TIA, 10 mg/kg; RET, 10 mg/kg; TET, 20 mg/kg; or CIP, 20 mg/kg. Larvae were observed daily for 5 days and percent of survival was calculated for each group.

### Statistical Analyses

Statistical tests were performed using GraphPad Prismv.5.04 (GraphPad Software Inc., San Diego, CA, United States). The *in vivo* survival data were plotted using the log rank test. *P*-value of ≤0.05 was considered significant.

## Results

### MICs of Antimicrobials against *S. aureus*

The MICs of study antibiotics against *S. aureus* strains are showed in **Table 2**. The MICs VAL, TIA, and RET on ATCC 29213, ATCC 43300, N9, and N54 were ranged from 0.03125 to 0.5 mg/L. The MICs of TET against the two ATCC strains were 0.5 mg/L which are considered susceptible by CLSI guidance. However, the two study clinical *S. aureus* strains were resistant to TET with MICs of 64 mg/L based on the CLSI break point (CLSI, 2015).

**Table 2:** The minimum inhibitory concentrations (MICs) of antibiotics against *Staphylococcus aureus* strains.

	MICs (mg/L)			
Antibiotics	ATCC 29213	ATCC 43300	N54	N9
CTX	4	16	4	16
ERY	0.25	0.25	0.25	0.25
FFC	8	8	8	8
CLI	0.25	0.125	0.125	1
CIP	0.25	0.5	0.5	0.5
ENR	0.125	0.125	0.25	0.25
GEN	0.5	1	0.5	0.5
AMK	1	4	1	4
TET	0.5	0.5	64	64
VAL	0.0625	0.0625	0.0625	0.0625
TIA	0.5	0.5	0.5	0.5
RET	0.0625	0.03125	0.0625	0.0625
VAN	1	1	1	1
BCR	64	64	32	64
SMZ	128	>256	>256	>256
TMP	4	>256	>256	>256

*CTX, cefotaxime; ERY, erythromycin; FFC, florfenicol; CLI, clindamycin; CIP, ciprofloxacin; ENR, enrofloxacin; GEN, gentamicin; AMK, amikacin; TET, tetracycline; VAL, valnemulin; TIA, tiamulin; RET, retapamulin; VAN, vancomycin; BCR, bacitracin; SMZ, sulfamethoxazole; TMP, trimethoprim; ATCC 29213 and N54: MSSA; ATCC 4330 and N9: MRSA.*

### FICI Determination

The FICI of study antibiotic combinations are shown in **Table 3**. For all study strains, the FICI of the VAL/TET and RET/TET combinations were 0.375–0.5 indicating synergy effects. Tiamulin/tetracycline combination showed synergistic action against *S. aureus* ATCC 29213 strain, but additivity effect on ATCC 43300 and the two clinical strains with FICI of 0.75. While the FICI of VAL, TIA, RET in combination with CIP, or ENR were 4 or 5 that demonstrate antagonistic effects. Moreover, the interactions of other antibiotic combinations with pleuromutilins resulted in indifference with the FICI of 1.5 or 2 (data not shown for TIA and RET in combination with the other antibiotics).

**Table 3:** The fractional inhibitory concentrations index (FICI) of the combinations of antimicrobial agents against *Staphylococcus aureus*.

Antibiotics	FICI				Interaction
combination	ATCC 29213	ATCC 43300	N54	N9	
VAL+CTX	2	2	2	2	Indifferent
VAL+ERY	2	2	2	2	Indifferent
VAL+FFC	1.5	1.5	2	2	Indifferent
VAL+CLI	2	2	2	2	Indifferent
VAL+CIP	5	5	4	5	Antagonistic
VAL+ENR	5	5	4	5	Antagonistic
VAL+GEN	2	2	2	2	Indifferent
VAL+AMK	2	2	2	2	Indifferent
VAL+TET	0.375	0.5	0.5	0.5	Synergistic
VAL+VAN	2	2	2	2	Indifferent
VAL+BCR	1.5	2	2	2	Indifferent
VAL+SMZ	2	2	2	2	Indifferent
VAL+TMP	2	2	2	2	Indifferent
TIA+TET	0.5	0.75	0.75	0.75	Synergistic or additivity
RET+TET	0.5	0.5	0.5	0.5	Synergistic
TIA+CIP	4	4	4	4	Antagonistic
TIA+ENR	4	4	4	4	Antagonistic
RET+CIP	4	4	4	4	Antagonistic
RET+ENR	4	5	5	5	Antagonistic

*CTX, cefotaxime; ERY, erythromycin; FFC, florfenicol; CLI, clindamycin; CIP, ciprofloxacin; ENR, enrofloxacin; GEN, gentamicin; AMK, amikacin; TET, tetracycline; VAL, valnemulin; TIA, tiamulin; RET, retapamulin; VAN, vancomycin; BCR, bacitracin; SMZ, sulfamethoxazole; TMP, trimethoprim; ATCC 29213 and N54: MSSA; ATCC 4330 and N9: MRSA.*

### *In Vitro* Time-Killing Curves

**FIGURE 1:**
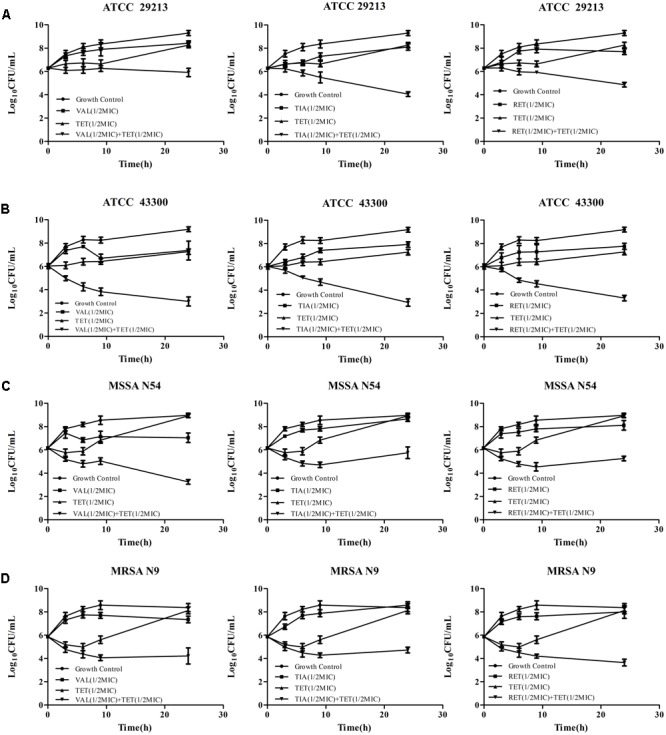
Time-kill curves of VAL, TIA, RET, and TET alone and in combinations against ATCC 29213 **(A)**, ATCC 43300 **(B)**, MSSA N54 **(C)**, and MRSA N9 **(D)**. Results show the mean ± standard error from three independent experiments.

The *in vitro* time-kill activities of the combination of pleuromutilins with TET at concentrations of 0.5 × MIC against study *S. aureus* strains are shown in **Figure 1** and specific log_10_ CFU/mL changes are shown in **Table 4**. All three study pleuromutilins in combination with TET had synergy effects against all study ATCC and clinical *S. aureus* strains. For instance, the combination of VAN with TET caused more than 2 log_10_ CFU/mL reductions on both MSSA and MRSA strains as compared with the most active antibiotic alone (**Figure 1** and **Table 4**).

**Table 4:** The log change (log_10_ CFU/mL) between the combinations vs. initial inoculum and the most active single agent after 24 h of incubation.

		Colony changes (log_10_ CFU/mL) at 24 h			
		ATCC 29213	ATCC 43300	N54	N9
VAL+TET	vs. initital inoculum	–3.37	–6.19	–5.71	–4.15
	vs. most active drug	–2.35	–4.26	–3.78	–3.13
TIA+TET	vs. initital inoculum	–5.24	–6.30	–3.21	–3.65
	vs. most active drug	–4.04	–5.03	–2.91	–3.41
RET+ TET	vs. initital inoculum	–4.44	–5.87	–3.71	–4.72
	vs. most active drug	–2.84	–3.94	–2.85	–4.35

*Valnemulin (VAL), tiamulin (TIA), retapamulin (RET), and tetracycline (TET). 1/2 × MICs of all antibiotics were used in this assay.*

### Optimal Inoculum Dose in the *G. mellonella* Model

A good infective dose-dependent survival rate was observed during 5 days post-infection time period in the *G. mellonella* model caused by the study *S. aureus* strains (**Supplementary Figures S1A–D** represent ATCC 29213, ATCC 43300, N54, and N9, respectively). At approximate 10^6^ CFU/larval infection dose, infected larval had 20–60% survival rates during experimental time period. At 10^3^–10^4^ CFU/larval infection doses, larval survival rates were 70–100% during the 5 days, while at approximate 10^8^ CFU/larval for ATCC 43300, and 10^9^ CFU/larval for other three *S. aureus* strains, animals dead within 24 h post-infection. Therefore, 10^6^ CFU/larval challenge dose was selected for the efficacy experiments in the model.

### Efficacy of Antibiotics for *G. mellonella*

**FIGURE 2:**
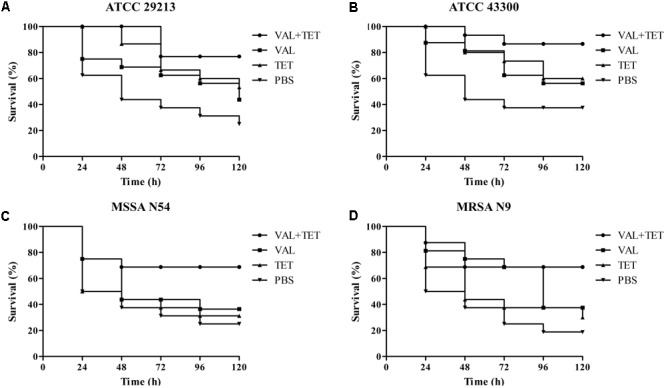
Survival rates of VAL and TET alone and in combination treatment in an experimental *G. mellonella* model caused by *S. aureus* strains ATCC 29213 **(A)**, ATCC 43300 **(B)**, MSSA N54 **(C)**, and MRSA N9 **(D)**.

**FIGURE 3:**
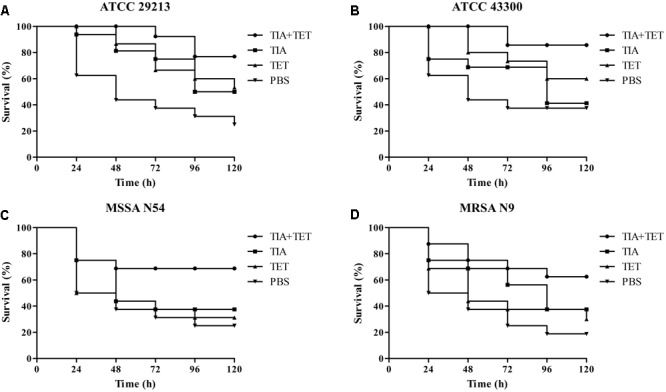
Survival rates of TIA and TET alone and in combination treatment in an experimental *G. mellonella* model caused by *S. aureus* strains ATCC 29213 **(A)**, ATCC 43300 **(B)**, MSSA N54 **(C)**, and MRSA N9 **(D)**.

**FIGURE 4:**
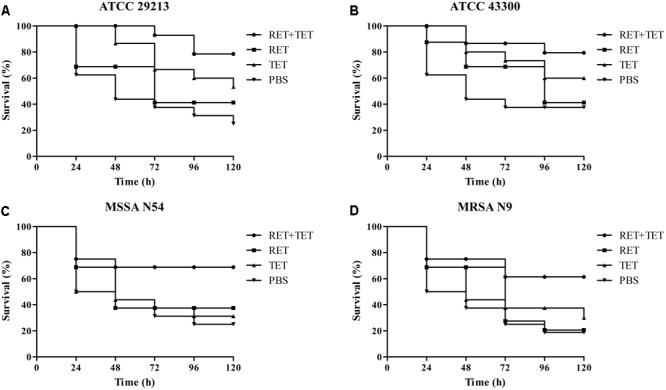
Survival rates of RET and TET alone and in combination treatment in an experimental *G. mellonella* model caused by *S. aureus* strains ATCC 29213 **(A)**, ATCC 43300 **(B)**, MSSA N54 **(C)**, and MRSA N9 **(D)**.

**FIGURE 5:**
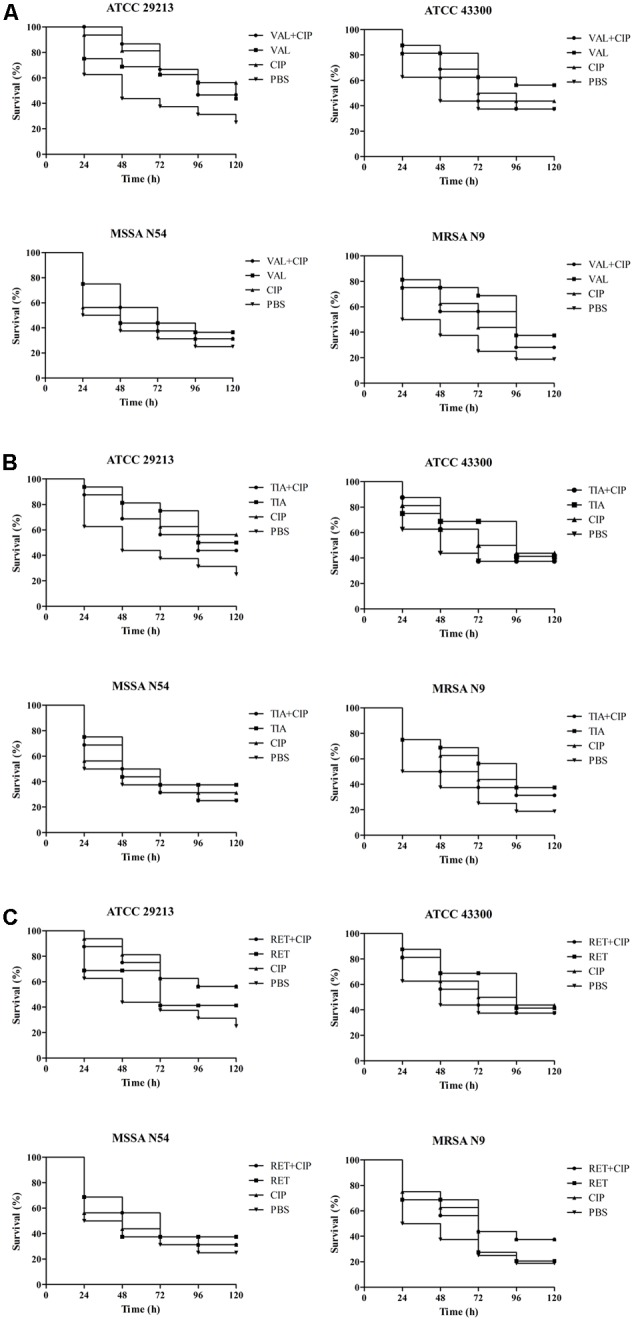
Survival rates of VAL **(A)**, TIA **(B)**, RET **(C)**, and CIP alone and in combination treatment in an experimental *G. mellonella* model caused by *S. aureus* strains ATCC 29213, ATCC 43300, MSSA N54, and MRSA N9.

The efficacies of pleuromutilins alone and in combination with TET against the four study *S. aureus* strains in the *G. mellonella* model were presented in **Figures 2–4** for VAL, TIA, and RET, respectively. In this study, VAL and TET monotherapy increased *G. mellonella* survival from infections caused by all study *S. aureus* strains. Importantly, the combination of VAL with TET significantly increased survival as compared with VAL and TET treatment alone in the model (**Figure 2**; *p* < 0.05). Similarly, the combinations of TIA/TET and RET/TET also significantly improved animal survival rates as compared with monotherapy (**Figures 3, 4**, respectively; *p* < 0.05). The efficacies of pleuromutilins alone and in combination with CIP against the four study *S. aureus* strains in the *G. mellonella* model were presented in **Figure 5**. The combinations of VAL/CIP, TIA/CIP, and RET/CIP did not improve the larvae survival rates as compared with monotherapy.

## Discussion

The emergence of multiple drug-resistant bacteria is a great threat to public health. Antibiotics combinations offer potential strategies to increase the therapeutic efficacy of antibiotics against infections caused by drug-resistant microorganism. Some recent studies have demonstrated that the combination of pleuromutilins derivative with doxycycline had synergy effect against multidrug-resistant *Acinetobacter baumannii in vitro* (Siricilla et al., 2017). Another investigation showed that TIA had a synergistic antimicrobial effect when in combination with chlortetracycline against *Mycoplasma* infection in birds (Islam et al., 2008). The present study was designed to study the anti-*S. aureus* activity of pleuromutilins (VAL, TIA, and RET) alone and in combination with other antibiotics with different mechanism of action both *in vitro and in vivo*.

We demonstrated that all study *S. aureus* strains, including MSSA and MRSA, had very low pleuromutilins MICs ranged from 0.03125 to 0.5 mg/L. In addition, the two study ATCC *S. aureus* strains are susceptible to TET, while the two clinical *S. aureus* strains were resistant to TET. Interestingly, synergistic effects of all study pleuromutilins, VAL, TIA, and RET, in combinations with TET were observed *in vitro* by a standard checkerboard methods and/or time-kill curves. However, antagonistic effects were exhibited in the combinations of pleuromutilins with two study fluoroquinolones (CIP and ENR). The exact mechanisms of these different interactions between pleuromutilins with other classes of antibiotic are not well identified. It is know that both pleuromutilins and TET belong to bacteriostatic agents. However, pleuromutilins and TET bind to bacterial 50S and 30S subunit of microbial ribosomes, respectively. Thus, the synergy effects between these two antibiotics might be due to their different bacterial targets and/or more complex relationships when they combined (Bollenbach, 2015). In consistence with other studies, we demonstrated that the combination of bacteriostatic with bactericidal antibiotics exhibited antagonism (e.g., pleuromutilins plus fluoroquinolones) or indifference effects (e.g., pleuromutilins in combination with most study bactericidal antibiotics) (Yeh et al., 2006; Ocampo et al., 2014). The mechanism of fluoroquinolones is to inhibit bacterial replication by blocking their DNA replication pathway (Bollenbach et al., 2009). On the other hand, pleuromutilins inhibit protein synthesis. Therefore, these different mechanisms of action might contribute to the antagonism effects between pleuomutilins and fluoroquinolones. However, the exact mechanism of these phenotypes is not clear up to date. In addition, it has been reported that these antagonism and indifference effects were antibiotics and/or organisms dependent (Pankey and Sabath, 2004; Ocampo et al., 2014).

Importantly, to the best of our knowledge, this is the first study demonstrated the synergy effects between pleuromutilins and TET in the experiment of *G. mellonella* infection model caused by MSSA and MRSA strains. Our results showed that the combination of VAL with TET significantly increased survival rates of animals infected by all study *S. aureus* strains as compared to the single treatment, with increased percent of survival from 30 to 90%. However, the combination of pleuromutilins with CIP do not increase survival rates of animals infected by all study *S. aureus* strains as compared to the single treatment, which is similar to the *in vitro* antagonistic effects by the checkerboard test. Recently, Desbois et al. used the same *G. mellonella* infection model due to *S. aureus* and demonstrated that anti-staphylococcal antibiotics, such as daptomycin and vancomycin, could increase larval survival (Desbois and Coote, 2011). In addition, penicillin improved survival of larvae infected with a penicillin-susceptible MRSA strain, but was ineffective with penicillin-resistant MRSA (Desbois and Coote, 2011). These results indicated that the *G. mellonella* model is useful for assessing the *in vivo* efficacy of anti-*S. aureus* agents.

## Conclusion

In the current studies, synergistic effects between pleuromutilins and TET were demonstrated both *in vitro* and in an experimental *G. mellonella* model caused by all four study MSSA and MRSA strains. There were no significant differences among the three pleuromutilins observed in *in vitro* assays, as well as in the *in vivo* animal mode. These findings provided important information that anti-staphylococcal effect of pleuromutilins is increased when it combined with TET. In addition, our study confirmed that the *G. mellonella* mode is a useful tool to investigate the *in vivo* efficacy of antimicrobial agents against *S. aureus* infections. We realized that our studies have some limitations. For instance, we only studied two ATCC and two clinical *S. aureus* strains. Future studies will include more MSSA and MRSA isolates. In addition, other animal models (e.g., murine bacteremia, skin and soft tissues infections) are needed to confirm the *in vivo* efficacy between pleuromutilins and other antibiotics. Moreover, we are interested in defining the mechanism of the antibiotic combined actions against *S. aureus*.

## Author Contributions

Y-HL and JS conceived this study and participated in its design and coordination. C-LD and YX designed the experiment and drafted the manuscript. C-LD, L-XL, and J-QL carried out the *G. mellonella* model experiments. S-WC, Z-HC, and YG carried out the time-kill curve studies and the checkerboard method. X-PL participated in the data analysis and revision of manuscript. All authors read and approved the final manuscript.

## Conflict of Interest Statement

The authors declare that the research was conducted in the absence of any commercial or financial relationships that could be construed as a potential conflict of interest.

## Supplementary Material

FIGURE S1 Kill kinetics of strains: ATCC 29213 **(A)**; ATCC 43300 **(B)**; MSSA N54 **(C)**; and MRSA N9 **(D)** at various numbers of CFU/mL in Galleria mellonella over 120 h.
